# Improved Configurational Sampling Protocol for Large Atmospheric Molecular Clusters

**DOI:** 10.1021/acsomega.3c06794

**Published:** 2023-11-13

**Authors:** Haide Wu, Morten Engsvang, Yosef Knattrup, Jakub Kubečka, Jonas Elm

**Affiliations:** Department of Chemistry, Aarhus University, Langelandsgade 140, 8000 Aarhus C, Denmark

## Abstract

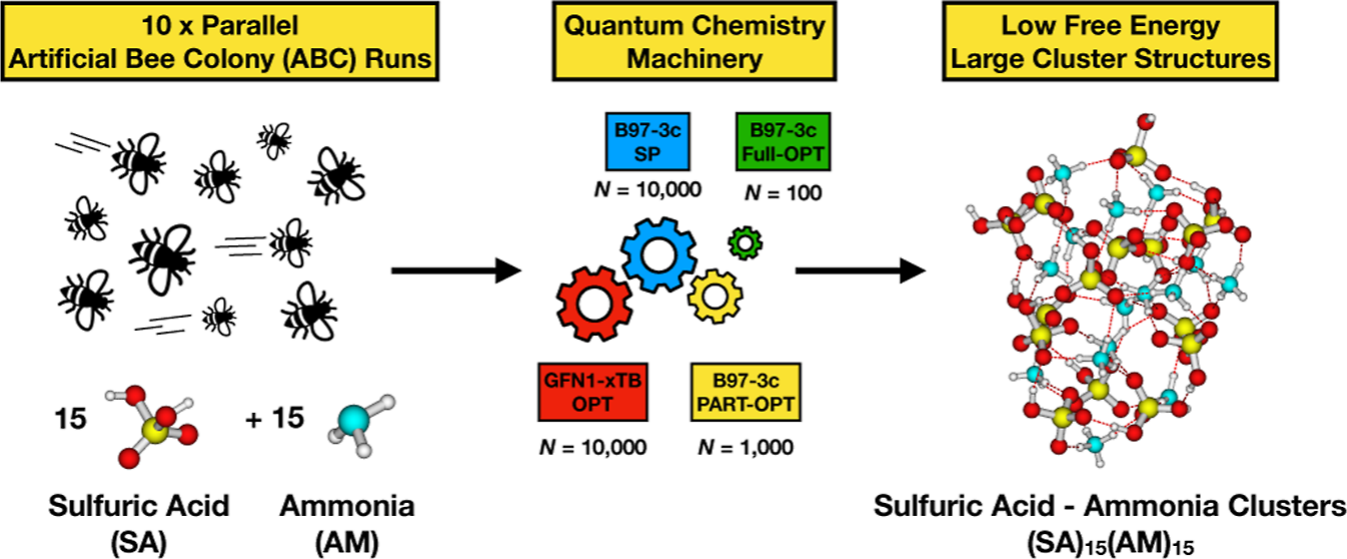

The nucleation process leading to the formation of new atmospheric particles plays a crucial role in aerosol research. Quantum chemical (QC) calculations can be used to model the early stages of aerosol formation, where atmospheric vapor molecules interact and form stable molecular clusters. However, QC calculations heavily depend on the chosen computational method, and when dealing with large systems, striking a balance between accuracy and computational cost becomes essential. We benchmarked the binding energies and structures and found the B97-3c method to be a good compromise between the accuracy and computational cost for studying large cluster systems. Further, we carefully assessed configurational sampling procedures for targeting large atmospheric molecular clusters containing up to 30 molecules (approximately 2 nm in diameter) and proposed a funneling approach with highly improved accuracy. We find that several parallel ABCluster explorations lead to better guesses for the cluster global energy minimum structures than one long exploration. This methodology allows us to bridge computational studies of molecular clusters, which typically reach only around 1 nm, with experimental studies that often measure particles larger than 2 nm. By employing this workflow, we searched for low-energy configurations of large sulfuric acid–ammonia and sulfuric acid–dimethylamine clusters. We find that the binding free energies of clusters containing dimethylamine are unequivocally more stable than those of the ammonia-containing clusters. Our improved configurational sampling protocol can in the future be applied to study the growth and dynamics of large clusters of arbitrary compositions.

## 1 Introduction

Based on the IPCC report,^1^ aerosol–cloud interactions are still the largest uncertainty in our understanding of our global climate. By aerosol photochemical properties^2^ and their ability to act as precursors for cloud condensation,^3^ these atmospheric particles play an important role. Approximately, half the number of cloud droplets are anticipated to be formed from newly formed aerosols.^4^ Hence, understanding the new particle formation (NPF) process of aerosol particles is crucial. NPF is thought to occur via the formation of atmospheric molecular clusters that potentially grow to larger sizes unless they are lost via coagulation with larger particles.^5^

A molecular cluster can be viewed as an aggregate of noncovalently bound molecules and as an intermediate between isolated molecules and a bulk system. The physicochemical properties of clusters are size-dependent and differ from bulk systems containing the same substances.^2,6^ By definition, microscale aerosol particles can, to a large extent, be considered bulk systems as the portion of surface molecules compared to the whole cluster becomes negligible with the increasing cluster size. The transition between clusters and bulk systems has not yet been unambiguously identified in aerosol research. Kulmala et al.^5^ partitioned the NPF process into three regimes and suggested the critical size for clustering to occur between 1.1 and 1.9 nm. While this may seem like a narrow range, the number of molecules in the cluster can range from a handful to hundreds in this size range. To date, no single technique is able to capture the entire route from single molecules to clusters, ending up as aerosol particles. However, advanced mass spectrometer techniques, such as the CI-APi-TOF,^7^ have been used to give insight into the chemical composition during clustering.

The properties of clusters within the cluster-to-particle transition regime are not well understood. In the context of NPF, the formation of atmospheric molecular clusters has been extensively studied using quantum chemical (QC) methods. In general, it has been found that sulfuric acid (SA) and bases, such as ammonia (AM)^8−10^ and alkylamines,^11−15^ form strongly bound clusters. The stability of SA–base clusters has been found to correlate with the basicity of the base for small cluster sizes.^16,17^ In addition, it has been found that SA–base clusters are most stable when they consist of an equal number of SA and base molecules, i.e., a 1:1 ratio.^18,19^ We refer to our recent reviews on organic^20^ and inorganic^21^ cluster formation for a comprehensive overview of studied cluster systems in the literature.

The largest cluster systems routinely studied using QC methods were limited to roughly eight molecules. In cluster dynamics simulations, it is inferred that cluster sizes larger than eight molecules are stable against evaporation. However, whether this is sufficient has not unambiguously been identified, and larger cluster systems are required to understand the cluster-to-particle transition regime. We recently pushed this limit by studying large (SA)_*n*_(AM)_*n*_ clusters with up to 60 molecules (*n* = 30).^22,23^ We identified that more exhaustive sampling methodologies, compared to the usual state-of-the-art sampling methodologies, might be required to accurately model such large clusters. Unfortunately, the computational cost increases exponentially with the growth of the system size. Unlike crystals or 2D periodic materials, large clusters are not stable periodic systems. In addition, the high complexity of their configurational space is caused not only by the cluster size but also by various cluster compositions.

A building-up computational approach could potentially reduce the computational cost. These approaches initially employ low-accuracy and less computationally demanding quantum mechanics (QM) methods combined with sampling algorithms to explore the potential energy surface (PES). Subsequently, more accurate and computationally expensive quantum chemistry methods are applied to obtain precise configurations. In such approaches, it is essential to establish correlations among the methods used at each step. The aforementioned difficulties present a bottleneck in the application of quantum chemistry to large clusters.

Here, we thoroughly assess computational protocols for sampling the configurational space of large atmospheric molecular clusters. We develop a significantly more accurate methodology and apply it to study large (SA)_*n*_(AM)_*n*_ and (SA)_*n*_(DMA)_*n*_ clusters, with *n* up to 15.

## 2 Methods

### 2.1 Computational Details

Semiempirical tight-binding calculations, with GFN1-xTB^24^ and GFN2-xTB,^25^ were performed using the XTB program.^26^ Calculations performed using the empirically corrected B97-3c,^27^ PBEh-3c,^28^ and r^2^SCAN-3c^29^ methods and DLPNO-CCSD(T_0_)^30,31^ calculations with normalPNO criteria^32^ were performed in ORCA 5.0.0.^33^ Calculations with the regular DFT functionals PW91, M06-2X, and ωB97X-D were performed using Gaussian16.^34^ Cluster configurational sampling was performed using the ABCluster program^35,36^ employing the CHARMM force field.^37^ Sampling using CREST 2.12^38−42^ was done with GFN1-xTB (--gfn 1) in noncovalent interaction mode (--nci) and with an energy threshold of 30 kcal/mol (--ewin 30). We did a quick ABCluster calculation to generate the initial structures (lm = 30, gen = 30, sc = 4, pop = 30) and optimized the structure using GFN1-xTB before being parsed to CREST. We used the lowest energy configuration as the “good guess” and the highest energy configuration as the “bad guess”. All the obtained cluster structures and thermochemistry have been added to the Atmospheric Cluster DataBase (ACDB).^43^

### 2.2 Cluster Binding Free Energies

We calculate the cluster binding free energies as the cluster free energy relative to the monomers; it is composed of
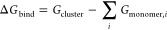
1

In cases where the method used for geometry optimization/vibrational frequency calculations differs from the calculation of the final binding energy, the free energy is calculated as
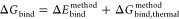
2

For instance, the geometry and vibrational frequencies can be calculated using cheaper computational methods given by the Δ*G*_bind,thermal_^method^ term. The binding energies can be calculated with more expensive and more accurate methods via the Δ*E*_bind_^method^ term.

The above equations consider only the thermochemistry of the clusters. We can calculate the binding free energies at given conditions as^44^
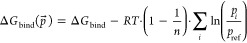
3where *p*_ref_ corresponds to a reference pressure (1 atm) and *p*_*i*_ represents monomer partial pressures. This equation, proposed by Wilemski and Wyslouzil,^45^ differs from some previous publications on cluster formation at “actual” conditions as these incorrectly generalized the unimolecular nucleation equation. Thus, eq 3 satisfies self-consistency also for multicomponent systems, i.e., having zero free energies of all monomers. Additionally, it fulfills the law of mass action which was previously violated by some other proposals.^46^

For large systems, numerous low vibrational frequency modes appear, which potentially leads to a large error in the calculated vibrational entropy contribution. We applied the quasi-harmonic approximation by Grimme^47^ to correct vibrational frequencies below 100 cm^–1^. In the quasi-harmonic approximation, these vibrations are treated as free rotors when calculating the vibrational entropy contribution. Unless otherwise stated, all calculations of free energies are presented at 298.15 K and a reference pressure of 1 atm.

### 2.3 Average Free Energies

In this work, we mainly focus on the SA–AM and SA–DMA systems consisting of the same number (*n*) of SA and base molecules. The intensive properties of a given cluster system should, with increasing cluster size, approach the properties of a bulk system. To gain insight into the intensive binding properties of clusters, we define the average binding quantity ( = Δ*G*_bind_/*n*) as the binding free energy per acid–base pair. The  is a measure of the average cluster stability. Also, it should converge to the free energy of an acid–base pair evaporation from its bulk system with a flat surface.

### 2.4 Construction of a Benchmark Set

To acquire a representative benchmark set for assessing the binding electronic energies of the (SA)_*n*_(AM)_*n*_ and (SA)_*n*_(DMA)_*n*_ clusters, we extracted the available cluster structures from the literature. The SA–AM clusters, with up to 6 SA and 6 AM, were taken from Besel et al.^48^ The SA–DMA clusters, with up to 4 SA and 4 DMA, and the SA–AM–DMA clusters, with up to 4 SA and 4 bases (AM or DMA), were taken from Myllys et al.^49^ Clusters with an equal number of acid and base molecules as well as clusters with one more acid or base molecule were considered in the test set. Including the monomers, this leads to a test set of a total of 44 structures. All the clusters were optimized at the ωB97X-D/6-31++G(d,p) level of theory, and high-level DLPNO–CCSD(T_0_)/aug-cc-pVTZ single-point energies, with a normalPNO criteria, were calculated on top of each of the cluster geometries.

The root-mean-square deviation (RMSD) between the calculated and reference geometries was utilized to evaluate the performance for obtaining the cluster structures. The RMSD was calculated using the ArbAlign program,^50^ which is a package for the most similar alignment of atomic coordinates between two molecular structures. The RMSD is calculated for each of the molecules in the benchmark set and is shown as an average. For evaluating the performance of energy calculation, we use mean absolute error (MAE) between all (*n*) molecules calculated at the reference (noted as “Ref.”) and at the calculation specific (noted as “Calc.”) methods
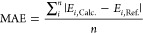
4

## 3 Results and Discussion

### 3.1 Benchmarking Small Systems

When modeling extremely large cluster systems, we must accept a decrease in the applied level of theory. However, we still need to ensure that the applied methods yield reliable results. In the recent study by Engsvang and Elm,^22^ we benchmarked the cluster structures and binding energies for the (SA)_*n*_(AM)_*n*_ clusters, with *n* = 1–6. Compared to the benchmark geometries, optimized at ωB97X-D/6-31++G(d,p), the GFN1-xTB method performed better than other semiempirical methods (PM6, PM7, and GFN2-xTB), providing similar cluster structures (RMSD = 0.31 Å) and thermal contributions to the free energy (MAE = 2.0 kcal/mol). It was also found that B97-3c yielded good agreement in binding energies with the benchmark DLPNO-CCSD(T_0_)/aug-cc-pVTZ level, with an MAE of 2.1 kcal/mol. Here, we extend this analysis to the SA–DMA and SA–AM–DMA systems as well as assess clusters with one more acid or base molecule in the clusters. In addition, compared to our previous study, we also test how the empirically corrected PBEh-3c, B97-3c, and r^2^SCAN-3c functionals perform for obtaining the geometries.

#### 3.1.1 Cluster Geometries

Using the constructed benchmark set, we evaluated how well different approximate methods resemble the benchmark geometries. All optimizations were initiated with reference geometry. Table 1 presents the average RMSD values between various methods and the reference geometries. It should be noted that the performance of the different methods varies for different systems in the benchmark set. Hence, no general trend can be observed in the RMSD patterns (see Figure S1 in the Supporting Information).

**Table 1:** Comparison between the Geometries Optimized by Different Methods for the SA–AM, SA–DMA, and SA–AM–DMA Systems^a^

method	mean RMSD/Å
Semiemp	
GFN1-xTB	0.34
GFN2-xTB	0.44
DFT-3c	
PBEh-3c	0.18
B97-3c	0.11
r^2^SCAN-3c	0.09
DFT/S	
PW91	0.19
M06-2X	0.25
ωB97X-D	- (ref)
DFT/L	
PW91	0.19
M06-2X	0.18
ωB97X-D	0.07

a The RMSDs (in Å) are calculated compared to the reference geometries given by Besel et al.^48^ and Myllys et al.^49^ The reference geometries are calculated at the ωB97X-D/6-31++G(d,p) level. S refers to the small 6-31++G(d,p) basis set and L refers to the larger 6-311++G(3df,3pd) basis set.

It should be noted that all of the RMSDs are in general quite low. Hence, all of the methods locate the same minimum configuration. The semiempirical GFN1-xTB and GFN2-xTB methods show the largest RMSDs, with values of 0.34 and 0.44 Å, respectively. Hence, based on these findings, if a semiempirical method should be used in the funneling approach, GFN1-xTB is a better choice than GFN2-xTB. This is consistent with recent benchmark studies^22,23,51,52^ and shows that the trend follows here. The empirically corrected DFT methods, PBEh-3c, B97-3c, and r^2^SCAN-3c, perform well with RMSDs of 0.18, 0.11, and 0.09 Å, respectively. This indicates that B97-3c and r^2^SCAN-3c could be good choices for obtaining accurate geometries at a lower cost. Interestingly, the two other DFT methods, PW91 and M06-2X, are in all cases worse than the DFT-3c methods, even when using large basis sets. Employing the larger 6-311++G(3df,3pd) basis set with the ωB97X-D functional yields a geometry very similar to that utilizing the smaller 6-31++G(d,p) basis set. This further illustrates that the geometries are not that dependent on the employed basis set but more on the functional.

#### 3.1.2 Binding Energies

**Figure 1:**
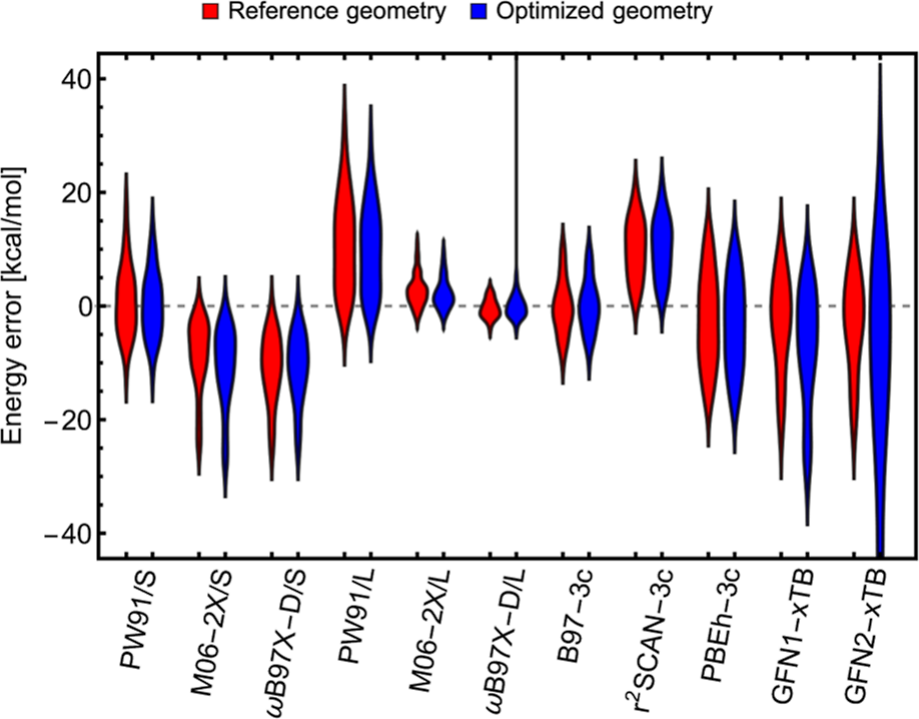
Error in binding energies calculated for the optimized and reference geometries. S and L refer to the 6-31++G(d,p) and 6-311++G(3pd,3df) basis sets, respectively.

The binding energies obtained by each method were benchmarked against DLPNO–CCSD(T_0_)/aug-cc-pVTZ calculations with the NormalPNO criterion, carried out on top of reference ωB97X-D/6-31++G(d,p) geometries. The mean absolute error of these results is presented in Figure 1, where “reference geometry” refers to geometries optimized at ωB97X-D/6-31++G(d,p), and “optimized geometry” refers to geometries optimized with the same methods utilized for calculating the binding energies. Hence, the “optimized geometry” illustrates both the error in the binding energies and changes in the geometry.

For the DFT methods with the small 6-31++G(d,p) basis, PW91 performs significantly better than M06-2X and ωB97X-D. However, this is reversed for the larger 6-311++G(3df,3pd) basis set. This could indicate that substantial cancellation of errors is present in the PW91/6-31++G(d,p) calculations. Interestingly, the PBEh-3c and r^2^SCAN-3c methods, which did well in reproducing the geometries, present large errors in the binding energies. As expected, the semiempirical methods, GFN1-xTB and GFN2-xTB, exhibited poor performance in calculating binding energies, especially when calculating energy on top of geometries optimized by the same method. Our results indicate that B97-3c represents a good compromise between accuracy and efficiency and is expected to be suitable for larger systems. If better accuracy is required, ωB97X-D/6-311++G(3df,3pd) or coupled cluster binding energies are needed. However, these methods are extremely expensive for large clusters. These results are consistent with our recent studies^22,23^ and illustrate that the benchmark findings are most likely transferable to other SA–base systems as well.

Previous sections conclude that the B97–3c functional reproduces well the benchmark ωB97X-D/6-31++G(d,p) geometries and shows relatively low errors in the binding energies. Hence, it could be a cost-efficient method for obtaining the final free energies of large atmospheric molecular clusters.

### 3.2 Evaluating ABCluster Sampling

The previous study by Engsvang et al.^23^ indicated that modeling the growth of large SA–AM clusters could present large errors due to insufficient cluster configurational sampling. We further explore here the utilization of different methodologies for sampling large SA–base clusters using the (SA)_10_(AM)_10_ and (SA)_10_(DMA)_10_ clusters as test cases.

#### 3.2.1 Monomer Ionization

**Figure 2:**
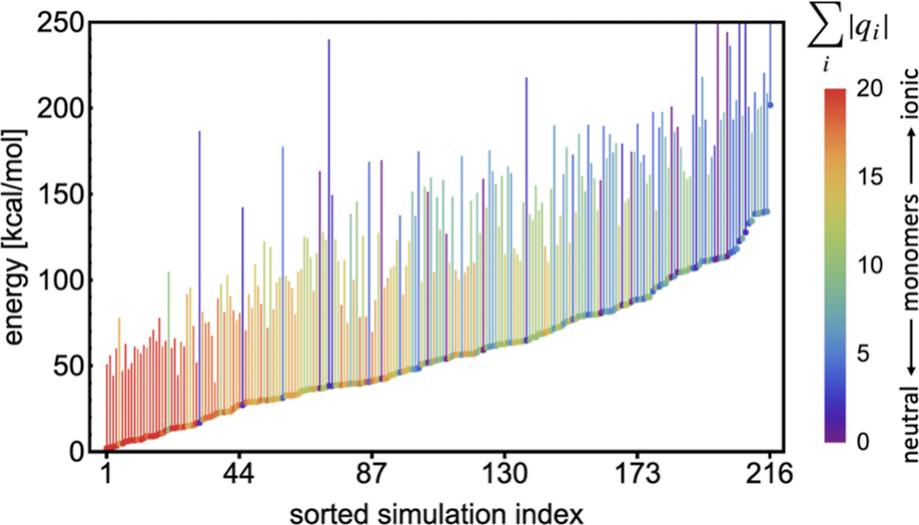
Distribution of GFN1-xTB energies (lines) with the lowest energy highlighted (points) for 216 possible monomer unit combinations forming the (SA)_10_(AM)_10_ cluster. The color represents the portion of ionic vs neutral monomeric units.

In our previous studies^22,23^ on large (SA)_*n*_(AM)_*n*_ clusters, we exclusively used ionic monomers in the ABCluster configurational sampling (CS) as proton transfer occurs in all SA–base clusters larger than 2 acid–base pairs.^8,11,18,19,48,49^ To verify that this is a reasonable assumption, we performed CS (lm = 2000, gen = 1000, sc = 4, pop = 300) for all 216 possible combinations of the acid monomeric unit (*trans*-H_2_SO_4_, *cis*-H_2_SO_4_, HSO_4_^–^, and SO_4_^2–^) and the base monomer unit (NH_3_ and NH_4_^+^) which lead to the overall-neutral (SA)_10_(AM)_10_ cluster. All structures are subsequently reoptimized at the GFN1-xTB level of theory to allow the comparison between differently built clusters as the ABCluster force-field energy would provide only the interaction energy of the rigid molecules. Whether the cluster was built from ionic or neutral monomeric units can be presented via the sum of the monomer charges (*q*). Figure 2 shows that the CS of ionic clusters leads to significantly lower GFN1-xTB energies.

It is clear that we can focus on the construction of the clusters from ionic monomers and, thus, save enormous computational time. Therefore, we further perform CS only using fully ionic monomers.

#### 3.2.2 ABCluster Parameters

**Figure 3:**
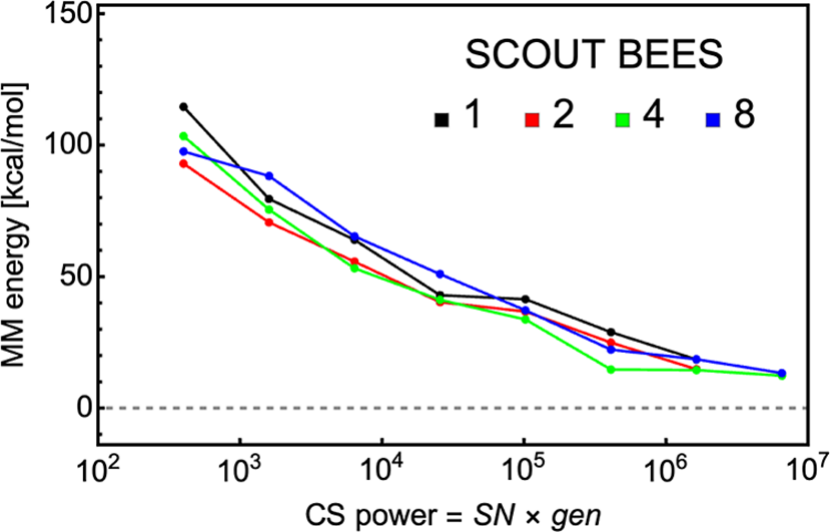
Average of global minimum energies over all ABCluster simulations with the same configurational sampling (CS) power, i.e., the product of the generations (gen) and population size (SN), gen × SN. The average is taken over simulations with the scout bee parameter giving four different sets of data (4 colored lines).

We performed five different ABCluster simulations for the ionic (SA)_10_(AM)_10_ cluster (i.e., using 10 bisulfate and 10 ammonium monomers) for each combination of the simulation parameters: the population size of SN ∈ (20, 80, 320, 1280, 5120), the number of generations (loops) of gen ∈ (20, 80, 320, 1280, 5120), and the maximal survival lifetime until replaced by another random structure *sc* ∈ (1, 2, 4, 8) (for more details regarding the ABCluster parameters, see the original papers^35,36^). In this case, the quality of the CS is evaluated by the lowest energy configuration found at the MM level. Figure 3 shows the average over all simulations with the same CS power and different *sc*.

It is seen that the quality of CS increases with increasing product gen × SN, which we will refer to as CS power. Hence, the exact choice of gen and SN parameters is not that important, and primarily, the CS power determines the quality of CS. However, we recommend a large enough population of at least SN = 100 to guarantee some level of diversity during the exploration. Similarly, the scout bee parameter *sc* shows only a little preference for the value of 4.

There is no set of parameters for which all simulations would find the global minimum. This is caused by the fact that for these large clusters the configurational space is very complex. The simulations get stuck in a tree branch of all energy minima if not diverse enough (i.e., small SN) and require significantly longer times to escape (i.e., large gen), or vice versa; for diverse ensemble (i.e., large SN), the simulations were too short (i.e., small gen) to explore the configurational space. Hence, the CS power would need to be significantly larger, but that would be computationally demanding. This is also the reason why, e.g., the long calculations with SN = gen = 5120 were not successfully finished. Nevertheless, we suggest circumventing this issue by running several parallel ABCluster runs. Based on the above findings, we chose the following parameters for each ABCluster simulation: SN = 1280, gen = 320, sc = 4, and saving 1000 lowest minima.

#### 3.2.3 Parallel ABCluster Runs

**Figure 4:**
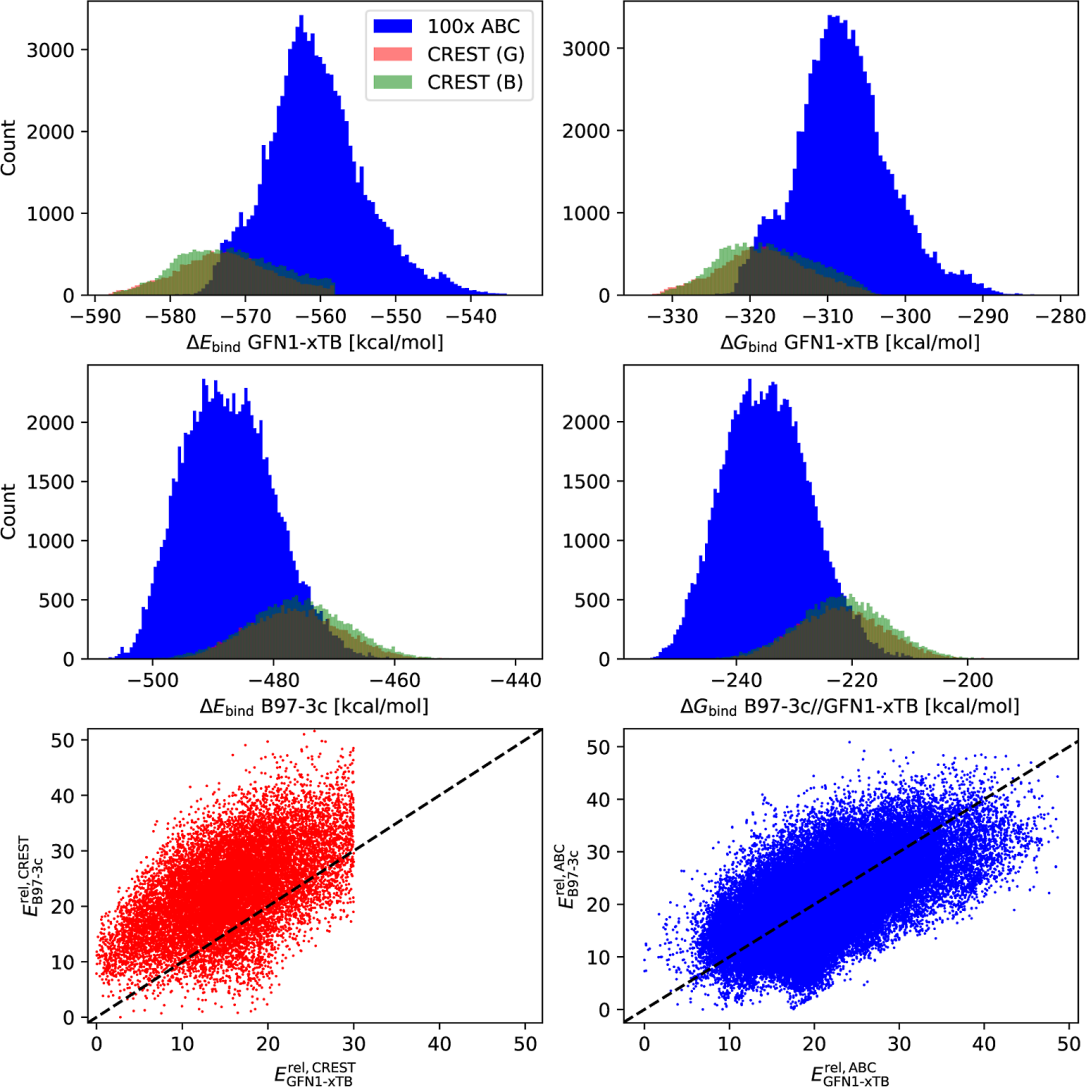
Distribution of the (SA)_10_(AM)_10_ binding energies/free energies based on 100 ABCluster runs vs a single CREST run with a bad (B) and a good (G) start guess.

To determine the optimal number of parallel runs, we conducted 100 parallel ABCluster runs on the (SA)_10_(AM)_10_ system using ionic monomers and the above-chosen parameters. All 1000 local minima for each run were optimized, and vibrational frequencies were calculated at the GFN1-xTB level of theory. B97-3c single-point energies were carried out on top of each cluster configuration. As a comparison, we also utilized CREST to search for cluster configurations. Figure 4 presents the distributions of the binding energies and binding free energies as well as the correlation between the GFN1-xTB and B97–3c binding energies for both sampling methods.

Both ABCluster and CREST yield a Gaussian-like distribution of the (free) energies. The distributions are unaffected by whether we evaluate the GFN1-xTB binding energies or the binding free energies (Figure 4, top panel). We find that CREST leads to lower (free) energy configurations compared to ABCluster. This is not surprising as the CREST calculations are sampled directly at the GFN1-xTB level, whereas ABCluster is sampled at the force-field level first and then optimized with GFN1-xTB.

The same conclusion can be drawn based on the B97-3c binding energies or the binding free energies calculated on top of the GFN1-xTB geometries. However, it is seen that the ABCluster and CREST distributions have reversed order (Figure 4, middle panel). This is surprising and implies that we cannot guarantee that screening at the GFN1-xTB level will yield meaningful data if the target level is B97-3c in the end. Previous studies on configurational sampling on small clusters using a funneling approach have found that GFN1-xTB is correlated to higher-level methods. Figure 4 in the bottom panel shows the correlation between GFN1-xTB and B97-3c. Unfortunately, little correlation is seen between the two methods, which implies that a meaningful cutoff cannot be applied at the GFN1-xTB level, and all configurations need at least single-point energy evaluations at the B97-3c level to ensure that low-energy conformers are not discarded.

Overall, we need many parallel runs to ensure that we are close to the global minimum. However, as the error in the binding energy of our B97-3c method is on the order of 3–4 kcal/mol, around 10 parallel ABCluster runs should be sufficient to yield errors that are below the method error (see Section S2 in the Supporting Information).

### 3.3 Extension to SA–DMA Clusters

To verify whether the number of parallel ABCluster runs (*N*_r_) and saved local minima (*N*_LM_) behaves differently for the (SA)_10_(DMA)_10_ system, we conducted four parallel series of calculations for comparison. Each series yielded 10,000 (*N*_r_ × *N*_LM_) local minimum structures. Subsequently, the 10,000 local minimum structures were further optimized by the GFN1-xTB method. Lastly, single-point calculations were conducted on the 10,000 GFN1-xTB optimized structures at the B97-3c level of theory.

**Figure 5:**
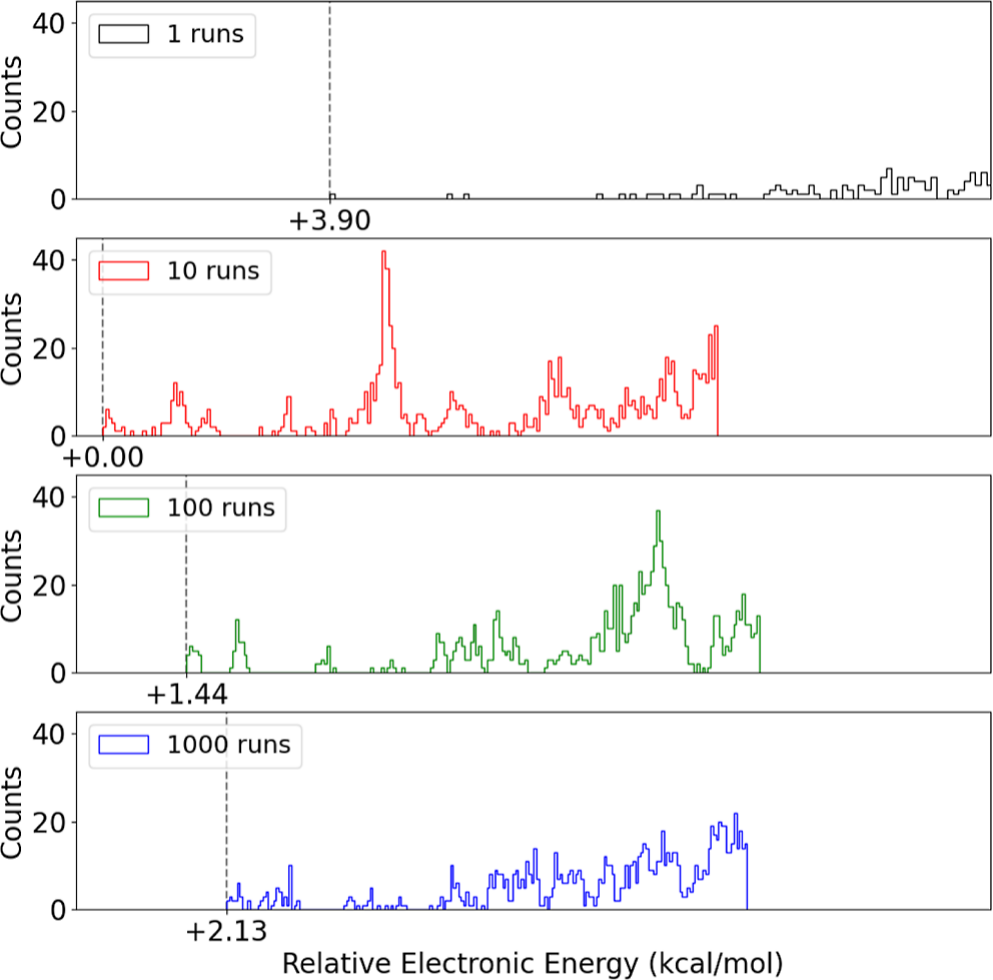
Distribution of (SA)_10_(DMA)_10_ electronic energies calculated at the B97-3c level of theory. Only the lowest 1000 calculations are shown in the histogram. The gray dashed line marks the lowest energies obtained (shown in kcal/mol).

Figure 5 displays the distribution of the 1000 lowest B97-3c energies of structures, calculated at the B97-3c level. The dashed line denotes the lowest energy conformer discovered. It is seen that increasing the number of runs while maintaining the *N*_r_ × *N*_LM_ constant does not significantly improve the ability to identify the global minimum. The test with (*N*_r_ = 10, *N*_LM_ = 1000) yielded the overall lowest-energy structure, while the minima of (*N*_r_ = 100, *N*_LM_ = 100) and (*N*_r_ = 1000, *N*_LM_ = 10) were 1.44 and 2.01 kcal/mol higher, respectively. As also confirmed in the previous section, using only a single run (*N*_r_ = 1, *N*_LM_ = 10,000) exhibited the poorest performance, with the obtained minima being 3.90 kcal/mol higher in energy. Again, it should be noted that the sampling errors for (*N*_r_ = 100, *N*_LM_ = 100) and (*N*_r_ = 1000, *N*_LM_ = 10) are still below the error of the applied B97-3c method for the binding energies. More runs would be more efficient if the same number of minima were saved for each simulation. However, this requires a lot of storage resources. Hence, for saving at max 10,000 minima (which are sufficient for studying SA–DMA), 10 runs with LM = 1000 seems to be the best choice.

#### 3.3.1 DFT-3c Energies Calculated on Top of GFN1-xTB Geometries

**Figure 6:**
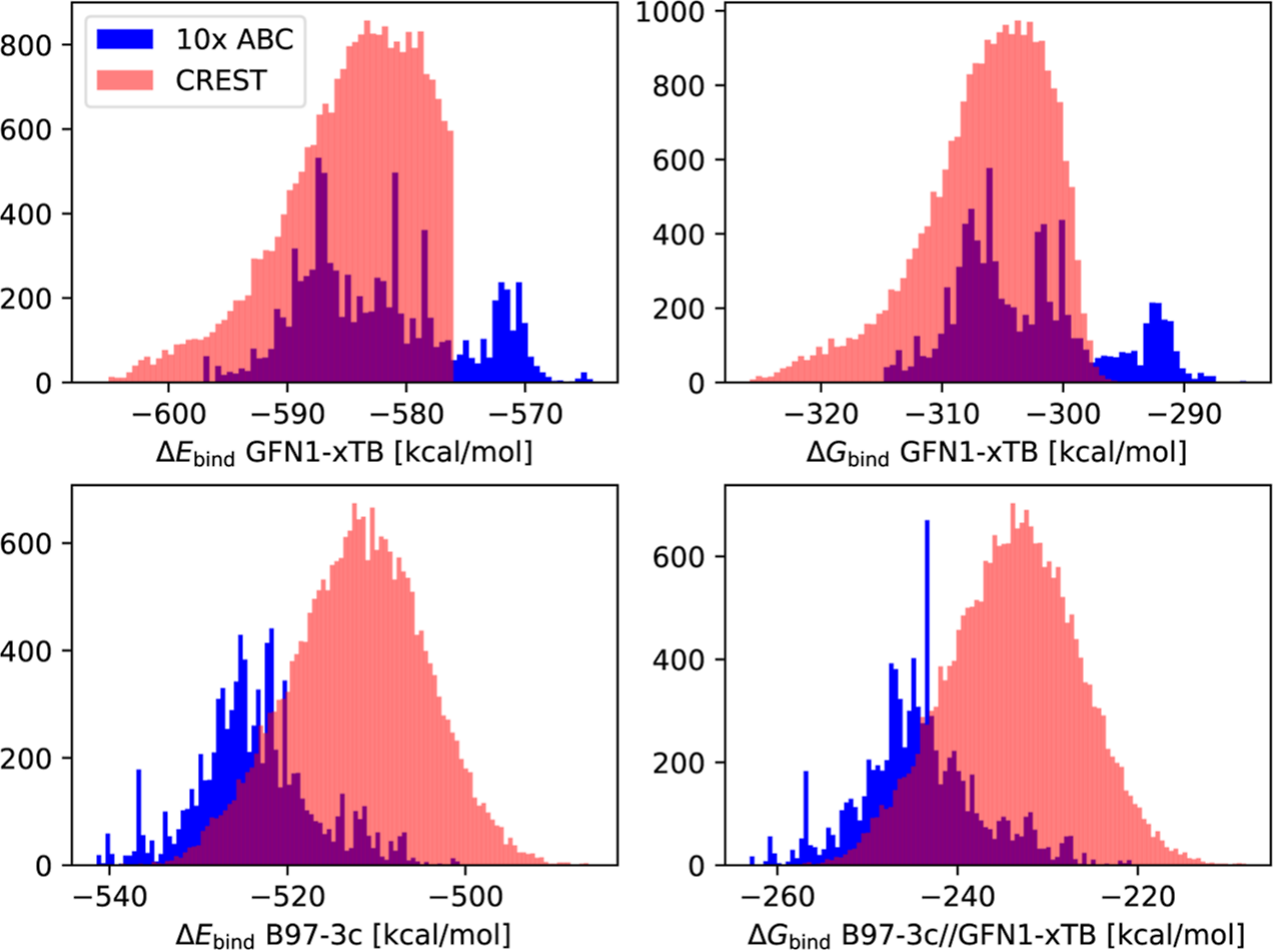
Distribution of the (SA)_10_(DMA)_10_ (free) energies of 10 ABCluster runs and a single CREST run.

We tested the distributions of the (SA)_10_(DMA)_10_ energies based on both ABCluster and CREST sampling. Using 10 parallel simulations, we obtained the same trends as those for the (SA)_10_(AM)_10_ system (see Figures 4 and 6).

We see a shift in the distribution similar to that shown for the (SA)_10_(AM)_10_ system. This further demonstrates that we cannot rely on the GFN1-xTB energies to obtain the best possible target B97-3c structures. Therefore, single-point calculations at the B97-3c level are required on top of all GFN1-xTB configurations.

#### 3.3.2 Geometries and Energies

**Figure 7:**
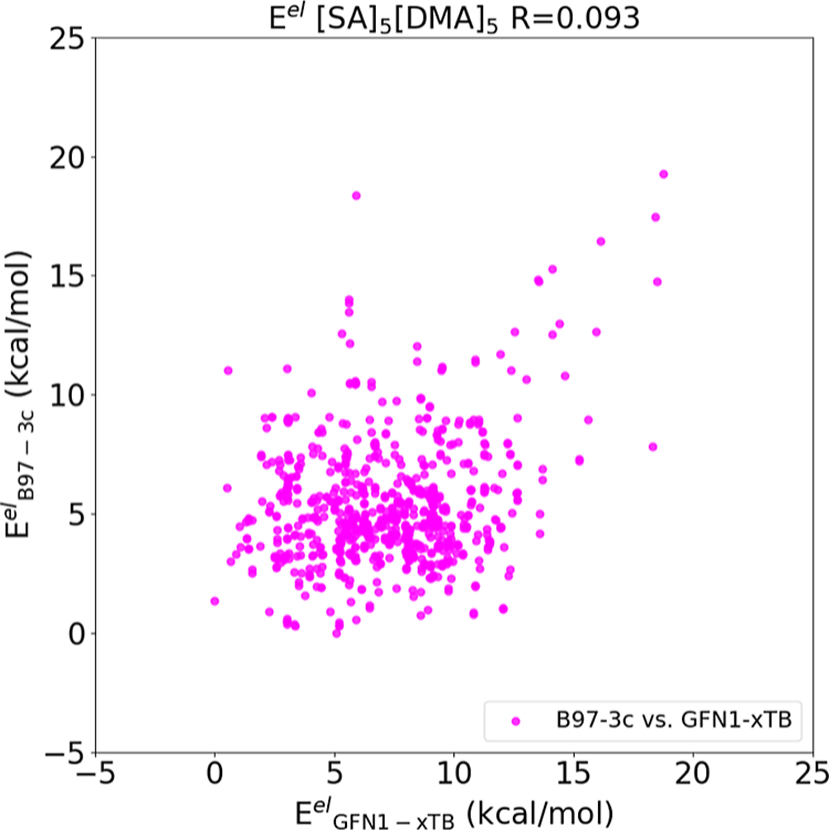
Correlation between the electronic energies of the (SA)_5_(DMA)_5_ cluster geometries optimized at the B97-3c, and GFN1-xTB level and plotted against each other. The energy values are relative with the lowest energy of each method set to zero.

Figures 4 and 6 indicate that there was little correlation between the GFN1-xTB (free) energies and the B97-3c (free) energies. We suspect this behavior to be caused by geometries that are too poor at the GFN1-xTB level of theory for large clusters, which are then too far away from the B97-3c target structures. A similar conclusion has previously been reached by Kurfman et al.^53^ For (SA)_3_ clusters, they found that all PM7 cluster configurations needed to be optimized at the DFT level to ensure that the lowest target structure was not missed. To look into this effect, we sampled the (SA)_5_(DMA)_5_ clusters with ABCluster and then fully optimized and calculated vibrational frequencies at the r^2^SCAN-3c and B97-3c levels, as well. Figure 7 presents the correlation of electronic energies for geometries optimized at B97-3c and GFN1-xTB. For each data point, the two geometry optimizations were initiated from the geometry given by ABCluster. Data for r^2^SCAN-3c can be seen in the Supporting Information, Figure S3.

Figure 7 shows that there is almost no correlation between the GFN1-xTB and B97-3c results (*R* = 0.093). This suggests that if our target method in the configuration sampling approach is B97-3c, we cannot rely on cutoffs in the GFN1-xTB (free) energies. Introducing such a cutoff would certainly lead to low-energy conformers being missed.

#### 3.3.3 Preoptimization by GFN1-xTB

The previous sections demonstrated that B97-3c and r^2^SCAN-3c have shown promising performance in generating optimized geometries, and both methods yield the most similar results. GFN1-xTB, being a low-level method, might not be accurate enough to produce the final structures. However, it can still be beneficial as a preoptimization method to save computational resources. We tested two different sampling schemes to investigate the potential computational gain in using GFN1-xTB for preoptimization of the (SA)_10_(DMA)_10_ cluster
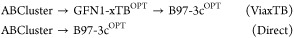


**Figure 8:**
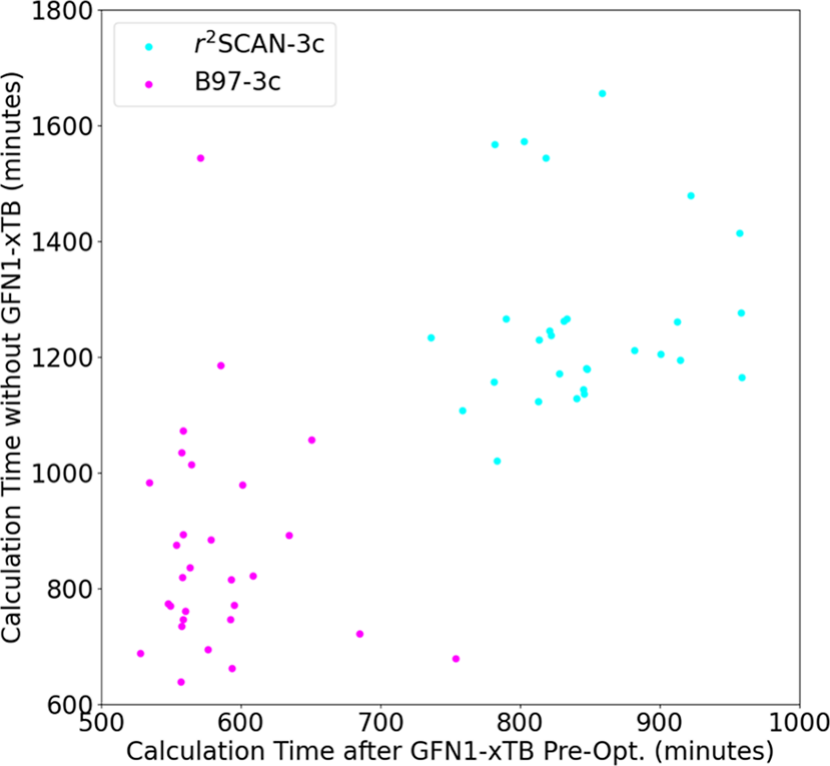
Run-time of 30 (SA)_10_(DMA)_10_ geometry optimizations at the B97-3c and r^2^SCAN-3c levels of theory initiated with ABCluster structures (*y*-axis, without GFN1-xTB preoptimization) and initiated with GFN1-xTB preoptimized structures (*x*-axis).

The preoptimization step should reduce the number of geometry cycles required to reach convergence at the B97-3c level, as the GFN1-xTB structures are still better than the output from the ABCluster force-field calculations. Figure 8 presents the timings of the two approaches tested on 30 (SA)_10_(DMA)_10_ cluster configurations.

Geometry optimization performed by GFN1-xTB takes only a few minutes. In contrast, B97-3c optimization takes between 10 and 25 h. However, this preoptimization significantly lowers the total run time of the DFT-3c methods by 30–50%. The preoptimization did not significantly alter the final B97–3c geometries, as shown in Figure S4. These findings show that while we cannot rely on the GFN1-xTB structures or energy for these large clusters, a massive amount of computational time is saved using GFN1-xTB as a preoptimization method. Figure 8 also shows that r^2^SCAN-3c takes relatively more time to finish compared to B97-3c. Considering that r^2^SCAN-3c is also less accurate in calculating energies (see Figure 1), B97-3c has been chosen for calculating the final results.

### 3.4 Energies vs Iterations

**Figure 9:**
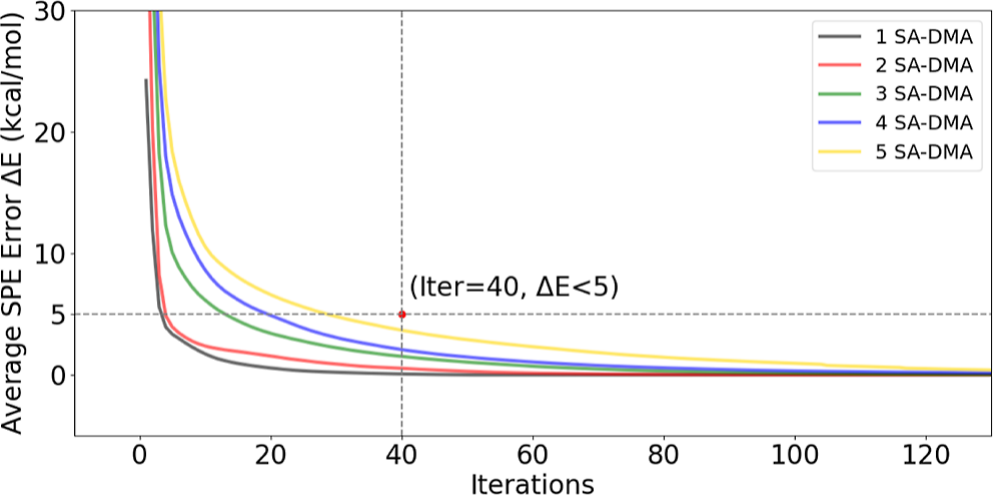
Estimated average of single-point energy (SPE) error as a function of the number of iterations during geometry optimization of the (SA)_*n*_(DMA)_*n*_ clusters, with *n* = 1–5. For each system, 1000 clusters were optimized at the B97-3c level of theory.

Fully optimizing tens of thousands of configurations at the B97-3c level of theory would be too computationally expensive. GFN1-xTB can reduce the computational time of the B97-3c optimization by giving a better start guess. During geometry optimization, the energy of the molecular structure decreases with each iteration as the geometry approaches convergence. Relaxing the convergence criteria can result in a reduction in the number of required iterations and can significantly expedite the optimization process. In the case of large cluster systems, the PES can exhibit significant complexity. In such instances, it is often advantageous to conduct a preliminary “pre-optimization” using the same method but with less stringent convergence criteria. This preoptimization step can aid in identifying and excluding configurations with high energies, thus enabling full optimization to be performed exclusively on conformers that exhibit lower energies and are more proximal to local minima. Figure 9 shows the geometry optimization convergence behavior of the (SA)_*n*_(DMA)_*n*_ systems, with *n* = 1–5. For each system, a total of 1000 full geometry optimizations were performed at the B97-3c level of theory. Each system is presented as the average over the entire ensemble of 1000 cluster configurations.

It is evident that the energy rapidly decreases during the first few iterations and subsequently slows. By the 40th iteration, the energy difference had decreased to below 5 kcal/mol. The 1000 calculations were indexed from 1 to 1000 and sorted by the final SPE from low to high. We find that after 10 iterations, the ordering of the calculations remains constant (see Figure S5). This suggests that after 10 iterations we can determine which calculations lead to low-energy local minima of the (SA)_5_(DMA)_5_ systems, enabling us to terminate the remaining calculations without overlooking potential local minima.

We tested the correlation of the energy between the fully optimized and partially optimized (20 optimization iterations) structures of the (SA)_5_(DMA)_5_ cluster. The partially optimized geometries are highly correlated (with *R* = 0.75) with those of the fully optimized geometries (see Figure S6). This finding supports the idea that we can shorten the geometry optimization process and identify potential candidates for global minimum after a certain number of iterations. Based on the above findings, we stopped the geometry optimization at 20 iterations, but for larger or more complex systems, it may be necessary to increase the number of iterations. Herein, we will apply a static number of 20 iterations. However, based on empirical evidence, we suggest a cutoff of 4*N* iterations in the future, where *N* refers to the number of acid–base pairs.

### 3.5 Building Up an Improved Configurational Sampling Approach

Based on the findings in the previous sections, we can now build up an improved configurational sampling approach that should be significantly more accurate than the previously applied methodologies. We suggest the following workflow
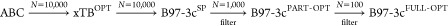


This approach begins with utilizing ABCluster to explore the PES and to search for low-energy conformers. A total of 10,000 local minima yielded by 10 parallel runs are saved as initial geometries for further optimization at the GFN1-xTB level. Due to the low reliability of energy calculations at the GFN1-xTB level and the impracticality of performing full geometry optimization at the DFT level for all 10,000 geometries, DFT single-point calculations are conducted on top of all the GFN1-xTB preoptimized geometries. Subsequently, filtering is performed based on a comparison of the energies calculated at the B97-3c level, resulting in the selection of 1000 low-energy conformers as candidates for leading us to the global minimum of the PES through further optimization. Next, 20 iterations of geometry optimization at the B97-3c level are conducted starting from these 1000 configurations. After this step, sorting the preoptimized structures based on their energies is expected to yield the same order as sorting the fully optimized results. Subsequently, another round of filtering is applied, retaining the 100 lowest energy conformers. Full geometry optimization is then performed starting from these 100 conformers. Finally, frequency calculations are carried out on top of these 100 optimized geometries to obtain the corresponding Gibbs free energies. The presented computational methodology should be applicable for studying large inorganic clusters containing the usual clustering of acid–base pair precursors. That is, it should work well for acids such as sulfuric, methanesulfonic, nitric, formic, iodic, and iodous in conjugation with bases such as AM, methyl/dimethyl/trimethylamine. Special care should be taken when studying flexible organic species to ensure that the rotational degrees of freedom of the monomers are adequately captured during cluster sampling.

### 3.6 Validation of the Methodology

To assess the performance of the outlined computational approach, we applied the workflow to study large clusters consisting of up to 15 SA–DMA and SA–AM pairs. Engsvang and Elm^22^ previously calculated the binding free energies of (SA)_*n*_(AM)_*n*_, with *n* up to 20, at the B97-3c level on top of the geometries optimized by GFN1-xTB. For a direct comparison, we fully optimized the 3 lowest free energy structures by Engsvang and Elm at the B97-3c level. In all cases, we found a lower free energy compared to that in our previous study, by as much as up to −12.9 kcal/mol. The only exception is the *n* = 10 cluster, where we found a structure 3.6 kcal/mol higher in free energy. Again, it should be noted that this is within the error margin of the applied B97-3c method. The (SA)_*n*_(DMA)_*n*_ (*n* = 2–8) clusters have previously been studied by DePalma et al.^10^ Again, we fully optimized the structures at the B97-3c level to allow a direct comparison. In all cases, we find significantly more stable clusters, up to −27.2 kcal/mol, for the (SA)_8_(DMA)_8_ cluster. Table S1 in the Supporting Information shows our improvements for the (SA)_*n*_(AM)_*n*_ (*n* = 6–15) and the (SA)_*n*_(DMA)_*n*_ (*n* = 2–8) systems in detail. This validates that our new approach is significantly more accurate than previously reported.

#### 3.6.1 SA–AM and SA–DMA Cluster Structures

**Figure 10:**
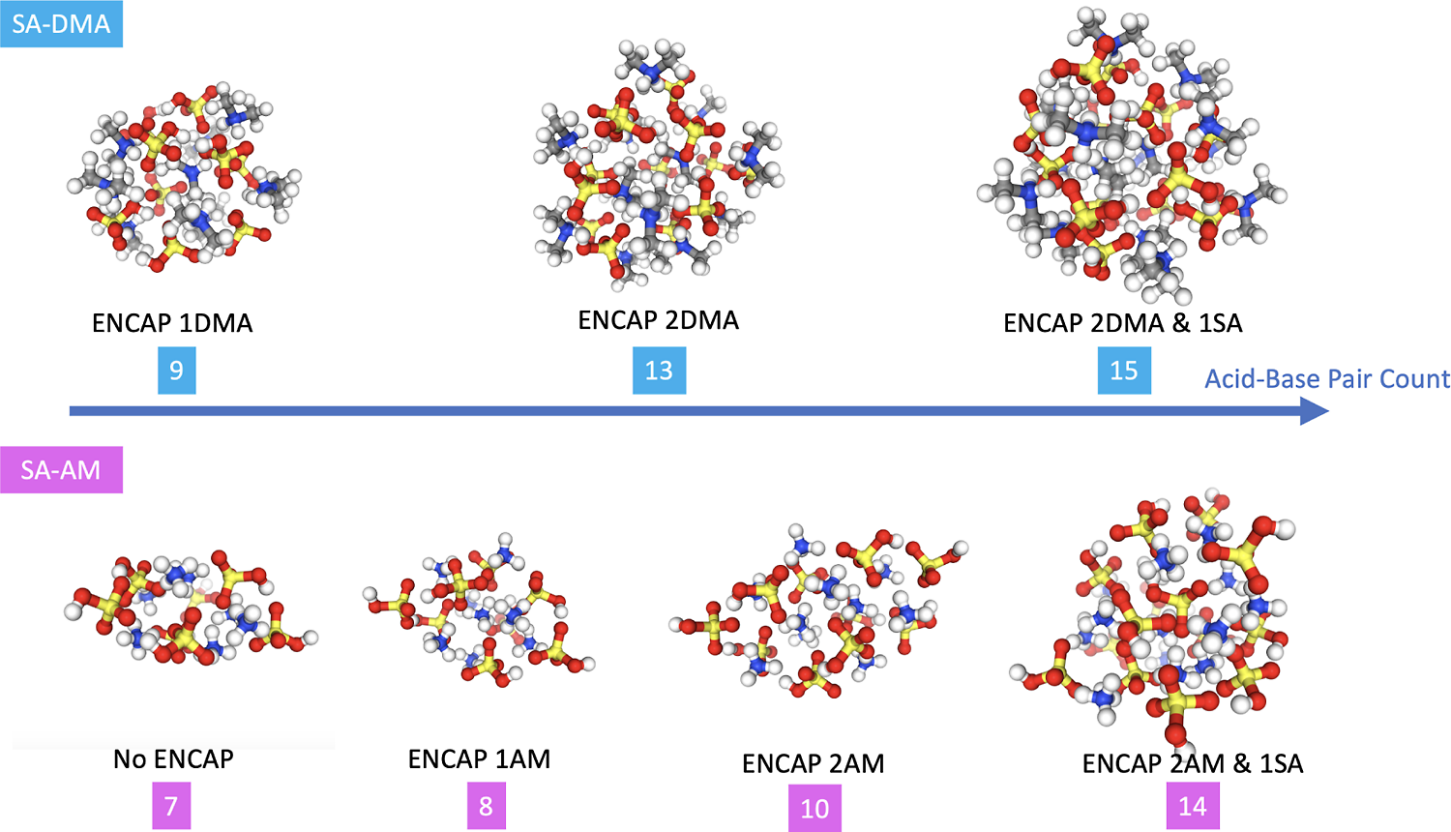
Structures of selected (SA)_*n*_(DMA)_*n*_ and (SA)_*n*_(AM)_*n*_ clusters (the numbers with color shade are the acid–base pair counts of clusters above; “ENCAP” stands for “encapsulated structure(s)”; “ENCAP 2DMA” means that there are two encapsulated DMA ions in this structure).

Figure 10 presents some of the cluster structures obtained using the new sampling methodology. The clusters are fully optimized at the B97–3c level of theory.

In the previous study of the SA–AM clusters by Engsvang and Elm,^22^ it was found that an ammonium ion was encapsulated, i.e., fully coordinated to surrounding molecules, in the cluster at the (SA)_7_(AM)_7_ cluster size. Here, we find that this occurs first for the (SA)_8_(AM)_8_ cluster. It should be noted that this difference is caused by the different levels of theory used to obtain the structures (GFN1-xTB and B97–3c). Interestingly, our approach resulted in a (SA)_10_(AM)_10_ structure with two encapsulated AM ions, slightly higher in free energy compared to the structure reported previously,^22^ which featured a single encapsulated AM ion. This illustrates that while our new sampling approach is significantly more reliable, it is not perfect, and care should be taken for systems with many degrees of freedom.

In the case of the SA–DMA systems, a similar encapsulated structure is observed when the number of SA–DMA pairs exceeds nine. In larger systems, starting from 13 pairs, multiencapsulated DMA configurations can be found. This is a surprising trend as one would assume that the bulky methyl groups would lead to highly unstable structures when coordinated with the surrounding bisulfate ions. However, the majority of the DMA molecules are not encapsulated and, thereby, the methyl groups are predominantly situated at the outside of the cluster structure, giving some degree of core–shell structure.

Encapsulated SA structures also appear in larger clusters, first observed in the (SA)_14_(AM)_14_ and (SA)_15_(DMA)_15_ systems. This suggests that SA has a lower propensity for encapsulation in clusters compared with amininium or ammonium ions. Notably, as the cluster size increases, encapsulation eventually becomes more prevalent. However, it is important to note that an encapsulated structure is not always the most stable configuration.

The largest cluster studied here reaches a geometric diameter of almost 2 nm, implying that the outlined methodology can be used to bridge the gap between theory and experiments.

#### 3.6.2 Binding Free Energies

**Figure 11:**
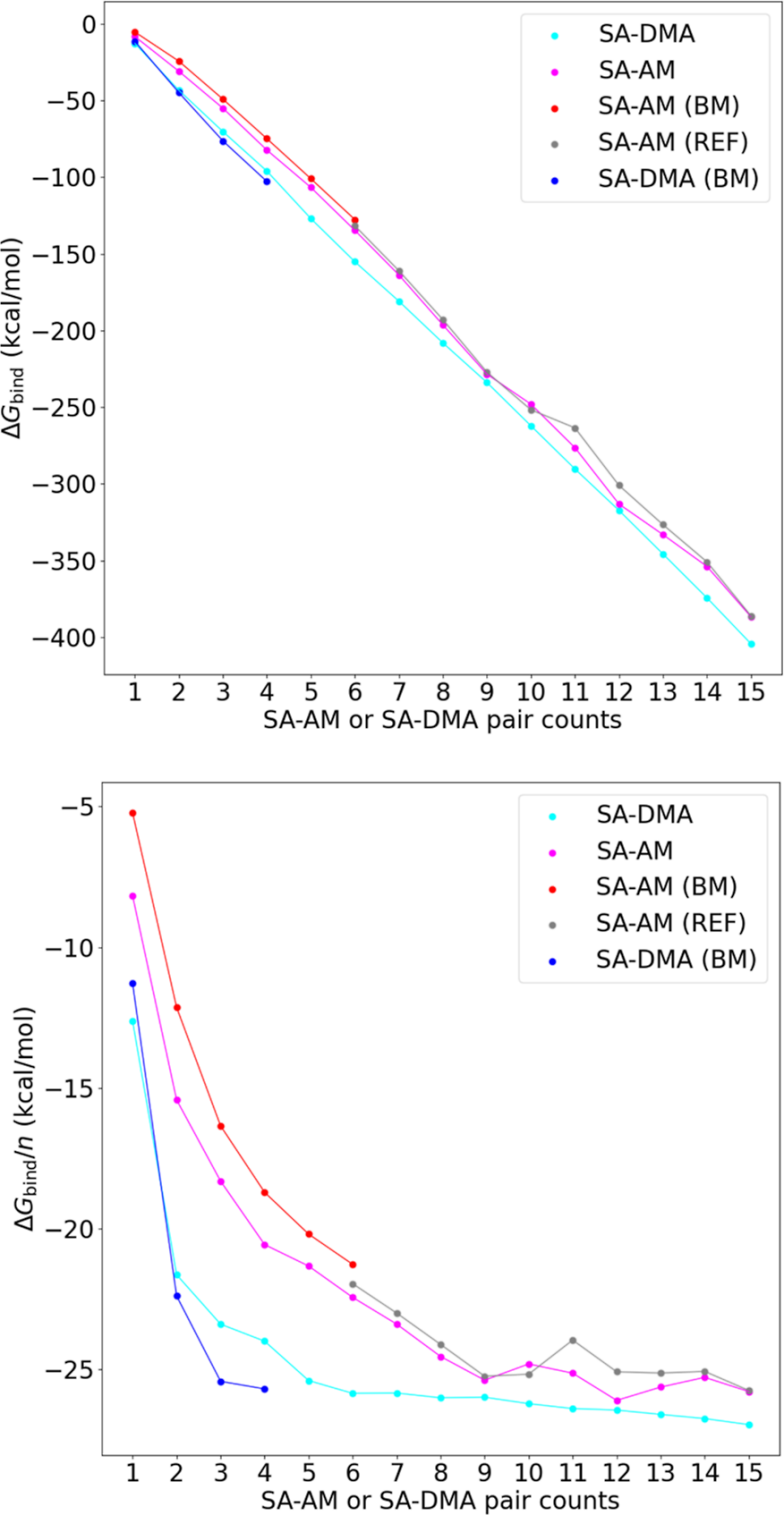
Binding free energies of (SA)_*n*_(DMA)_*n*_ and (SA)_*n*_(AM)_*n*_ clusters, *n* = 1–15 (top). Average binding free energy contribution from each SA–DMA or SA–AM pair in the clusters (bottom). (BM: benchmarking data calculated at the DLPNO-CCSD(T_0_)/aug-cc-pVTZ//ωB97X-D/6-31++G(d,p) level, (SA–AM) data were reported by Besel et al.;^48^ REF: reference data calculated at the B97-3c level reported by Engsvang and Elm.^22^)

Figure 11 presents the total binding free energy (left) and the average binding free energy contribution from each SA–DMA or SA–AM pair in the clusters (right). As a comparison, we also plotted the data of the SA–AM clusters reported by Besel et al.^48^ and the SA–DMA clusters reported by Myllys et al.^49^ These are calculated at the DLPNO-CCSD(T_0_)/aug-cc-pVTZ//ωB97X-D/6-31++G(d,p) level of theory, and the data are denoted as the “BM” series in the figures. As also pointed out in previous studies,^10,22^ the total binding free energy Δ*G*_bind_ decreases almost linearly as the cluster size increases. This is seen to be almost perfectly linear for SA–DMA, whereas there is a slight fluctuation observed for SA–AM. This is most likely due to the complexity of the SA–AM clusters, having a higher degree of freedom compared to the SA–DMA clusters.

The SA–DMA systems consistently exhibit lower free energies compared to the SA–AM systems. This is curious, as the encapsulation of an aminium ion should destabilize the clusters. However, this finding indicates that the preference for SA to bind more strongly to DMA compared to AM is retained, even for large clusters. One could speculate that mixed SA–AM–DMA clusters might be even more stable than the SA–DMA clusters by having an ammonium ion encapsulated in the core. DePalma et al.^10^ reported that SA–AM and SA–DMA have different preferences for hydration. While our study of dry clusters shows that SA–DMA is unequivocally more stable than SA–AM, hydration can be an interesting topic for further study.

The average binding free energies show that the SA–DMA clusters more rapidly reach an almost constant value compared to the SA–AM system. In addition, it is clear that the average binding free energy does not entirely level out but continues to slightly stabilize the cluster as it grows. We speculate that the average binding free energy reaching an almost constant value indicates that we are transitioning from discrete cluster configurations toward a dynamic continuum of cluster states. This hypothesis is backed by the encapsulation of ammonium/dimethylaminium ions, which begin to resemble a solution. For SA–AM, this occurs around 8–10 acid–base pairs, whereas for SA–DMA, it occurs already around 5–6 acid–base pairs.

#### 3.6.3 Free Energies at Given Conditions

**Figure 12:**
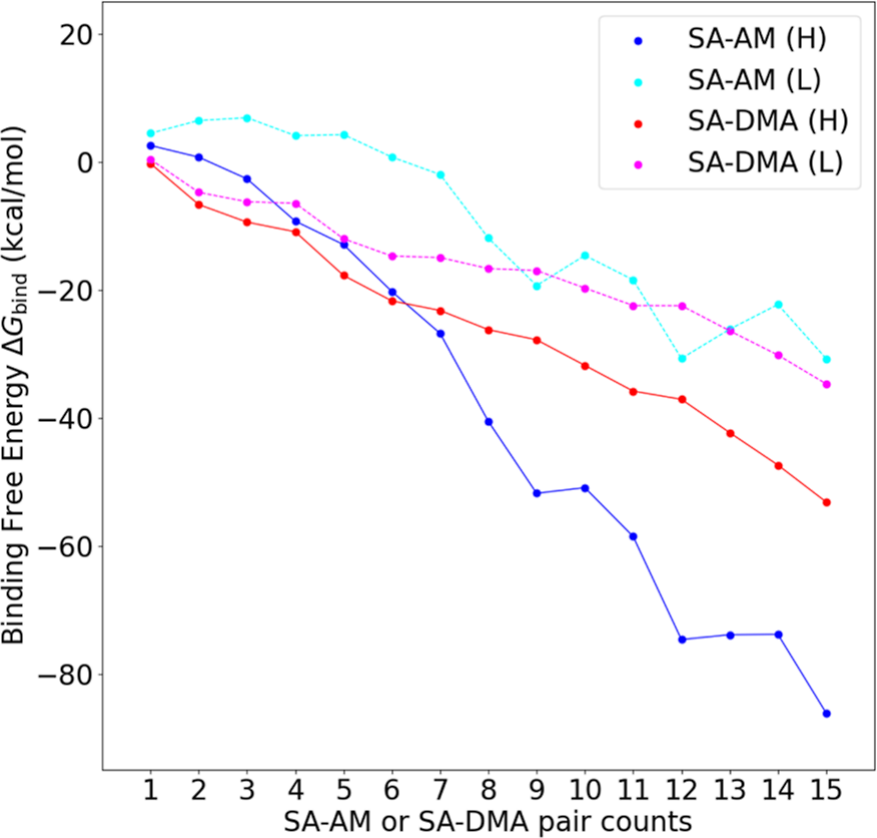
Binding free energies Δ*G*_bind_ of (SA)_*n*_(DMA)_*n*_ and (SA)_*n*_(AM)_*n*_ clusters (*n* = 1–15) at 278.15 K. With the high concentration of [SA] = 10^6^ molecules/cm^3^, [DMA] = 10 ppt [AM] = 10 ppb (denoted by “H” and solid lines) and low concentration of [SA] = 10^6^ molecules/cm^3^, [DMA] = 1 ppt, [AM] = 10 ppt (denoted by “L” and dashed lines).

Using the Gibbs free energies calculated above, it is possible to calculate the binding free energies under specific conditions of monomer concentrations and temperature. The self-consistent distribution function proposed by Wilemski and Wyslouzil^45^ was employed to establish the monomer free energies as zero. Figure 12 shows the binding free energies of the clusters at 278.15 K. This temperature was selected as it corresponds to typical CLOUD chamber measurements^54,55^ and observations of nucleation in the field. We studied a low-concentration regime ([SA] = 10^6^ molecules/cm^3^, [DMA] = 1 ppt, [AM] = 10 ppt) and a high-concentration regime ([SA] = 10^6^ molecules/cm^3^, [DMA] = 10 ppt, and [AM] = 10 ppb).

In all cases, cluster growth becomes favorable for larger clusters. For AM at 10 ppt, we see a slight free energy barrier. This barrier is suppressed at 10 ppb AM and the clusters form spontaneously. In both DMA cases ([DMA] = 1 or 10 ppt), we observed a barrier-free cluster formation process for the SA–DMA system. This is consistent with the CLOUD measurements, where SA–DMA nucleation is observed to occur at the collision limit.^56^ Overall, this illustrates that the usual assumption in cluster dynamics studies that the clusters are stable outside the eight-molecule area appears to hold for our current results. It is noteworthy that SA–AM (10 ppb) leads to lower free energies than SA–DMA (10 ppt) after *n* = 7. This could indicate that cluster growth will be dominated by AM instead of DMA at larger cluster sizes.

#### 3.6.4 Addition Free Energies

To model cluster growth, we added monomers to the (SA)_*n*_(base)_*n*_ clusters. However, we recently identified that the addition free energies were quite erratic for SA–AM clusters.^23^ This was concluded to be caused by insufficient sampling. Here, we recalculated the addition free energies for adding 1–2 SA molecules to the SA–AM clusters. These addition free energies correspond to the following reactions
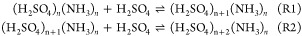


**Figure 13:**
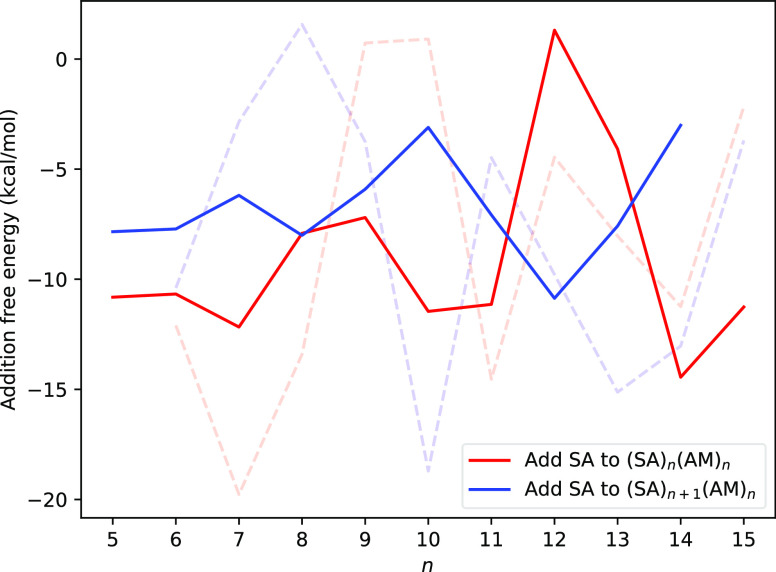
Addition free energies for adding one or two SA molecules to the (SA)_*n*_(AM)_*n*_ clusters at the B97-3c level of theory. The dotted lines represent the data from Engsvang et al.^23^

The calculated data are shown in Figure 13. The B97–3c//GFN1-xTB data from our previous study^23^ are plotted for comparison.

Compared to our previous studies,^23^ we see a significant improvement in the form of reduced scatter in the calculated addition free energies. However, despite the significantly improved sampling methodology presented here, we still observe an oscillatory behavior in the SA–AM addition free energies for clusters with more than 10 acid–base pairs. This can be caused by two reasons. Either the sampling methodology for “flexible” systems is still not accurate enough when we reach larger sizes of 10 or more SA–AM pairs, or when we reach 10 or more SA–AM pairs, the clusters cannot anymore be viewed as individual configurations and the addition free energies should be calculated over an ensemble of configurations.

## 4 Conclusions

This work introduces a systematic and comprehensive computational approach for exploring the configurational space of large atmospheric clusters far beyond the size routinely studied in the literature. We find that parallel ABCluster runs are required to achieve a good guess for low-energy cluster structures. In addition, we applied the B97–3c method for optimizing the geometries and calculating the vibrational frequencies.

Applying the improved sampling approach, we investigated the SA–AM and SA–DMA clusters containing up to 15 acid–base pairs. The largest clusters obtained reached a size of almost 2 nm, which is in line with the experimental detection limit of modern particle counters. We believe that this approach can be extended to larger or more complex systems by increasing the number of saved local minima or the maximum number of iterations.

In addition, we presented the structures and binding free energies of the SA–AM and SA–DMA clusters comprising up to 15 acid–base pairs. Interestingly, SA was found to have a lower priority for encapsulation compared with aminium or ammonium ions. It was also observed that encapsulated structures are not always the most stable configurations.

Overall, this study provides a computational approach that can be applied to large clusters of arbitrary compositions. In future work, we will apply this approach to improve our understanding of the composition of growing clusters. Such information is valuable for understanding the exact composition of freshly nucleated particles in the atmosphere.

## Supporting Information Available

Section S1: Plot of the RMSD patterns for the full benchmark set of 44 molecules. Section S2: distributions of the (SA)_10_(AM)_10_ cluster binding free energies from 100 ABCluster runs. Section S3: correlation between energies calculated by GFN1-xTB, r^2^SCAN-3c, and B97-3c. Section S4: geometries from calculations with and without preoptimization using GFN1-xTB. Section S5: number of iterations required for partial geometry optimization. Section S6: comparison of (SA)_*n*_(AM/DMA)_*n*_ low energy conformers to previous study (PDF)

## Notes

All the calculated structures and thermochemistry are available at: https://github.com/elmjonas/ACDB/tree/master/database_v2/Articles/wu23_sa_am_dma.
