# Correction to “Improved Configurational Sampling Protocol for Large Atmospheric Molecular Clusters”

**DOI:** 10.1021/acsomega.5c11445

**Published:** 2025-11-18

**Authors:** Haide Wu, Morten Engsvang, Yosef Knattrup, Jakub Kubečka, Jonas Elm

We identified a mistake in our code that calculates free energies under given conditions in Section 3.6.3 and generates Figure 12. This implies that the correct concentration of sulfuric acid written in the paper should be set to 10^8^ molecules cm^–3^ instead of 10^6^ molecules cm^–3^ to give the correct trends. This error has no impact on the main conclusions drawn in Section 3.6.3, and the primary focus of the paper about the development of an improved configurational sampling protocol is unaffected by the error.

The mistake leads to minor corrections in section 3.6.3. The barrier-free cluster formation process for the SA–DMA system should be corrected to only be observed at [DMA] = 10 ppt. In addition, the observation that SA–AM at 10 ppb leads to lower free energies than SA–DMA at 10 ppt should be corrected to occur after n = 6.

**Figure 12:**
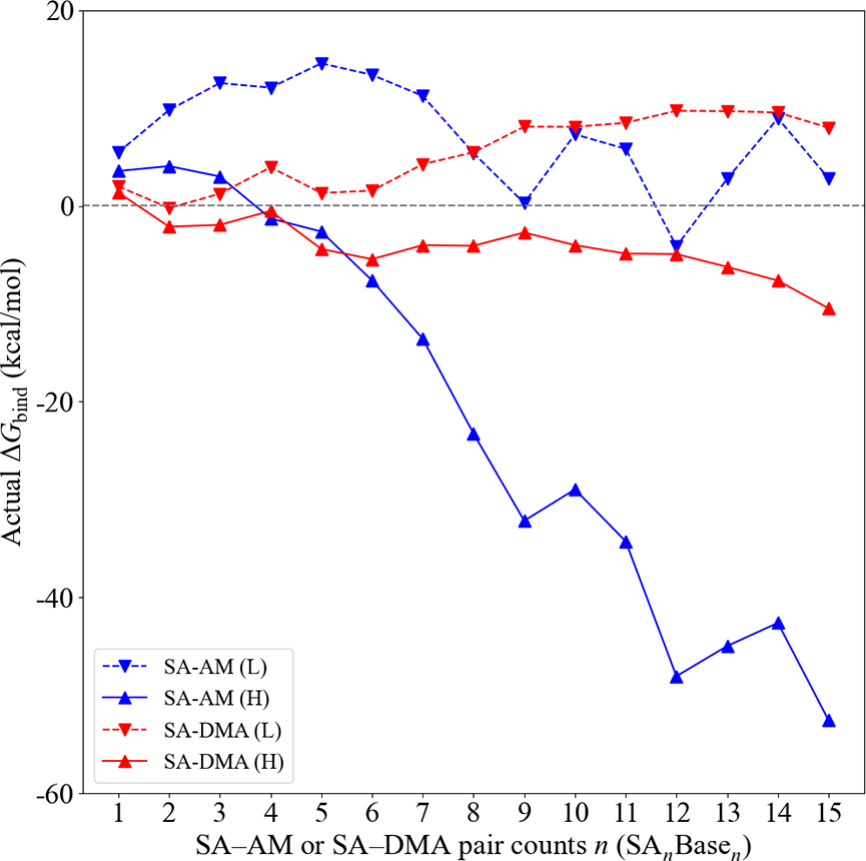
Binding free energies Δ*G*_bind_ of (SA)_*n*_(DMA)_*n*_ and (SA)_*n*_(AM)_*n*_ clusters (*n* = 1–15) at 278.15 K. With the high concentration of [SA] = 10^6^ molecules/cm^3^, [DMA] = 10 ppt [AM] = 10 ppb (denoted by “H” and solid lines) and low concentration of [SA] = 10^8^ molecules/cm^3^, [DMA] = 1 ppt, [AM] = 10 ppt (denoted by “L” and dash lines).

