# Supplementary material for: Improved Configurational Sampling Protocol for Large Atmospheric Molecular Clusters
Source: ACS Omega. 2023 Nov 13;8(47):45065–77. doi: 10.1021/acsomega.3c06794 (PMC10688134; doi:10.1021/acsomega.3c06794)
Supplement: Supplementary file 1 — ao3c06794_si_001.pdf [file ao3c06794_si_001.pdf]

Supporting Information

for

Improved Configurational Sampling Protocol for Large Atmospheric Molecular Clusters

Haide Wu, Morten Engsvang, Yosef Knattrup, Jakub Kubečka and Jonas Elm*

Department of Chemistry, Aarhus University, Langelandsgade 140, 8000 Aarhus C, Denmark

E-mail: jelm@chem.au.dk

S1 Comparison Between Optimized and Reference Geometries of 44 Molecules

Figure S1 shows the RMSD between optimized geometries and reference geometries of the 44 systems. The benchmarking method is ωB97X-D/6-31G++(d,p).

RMSD by methods.png

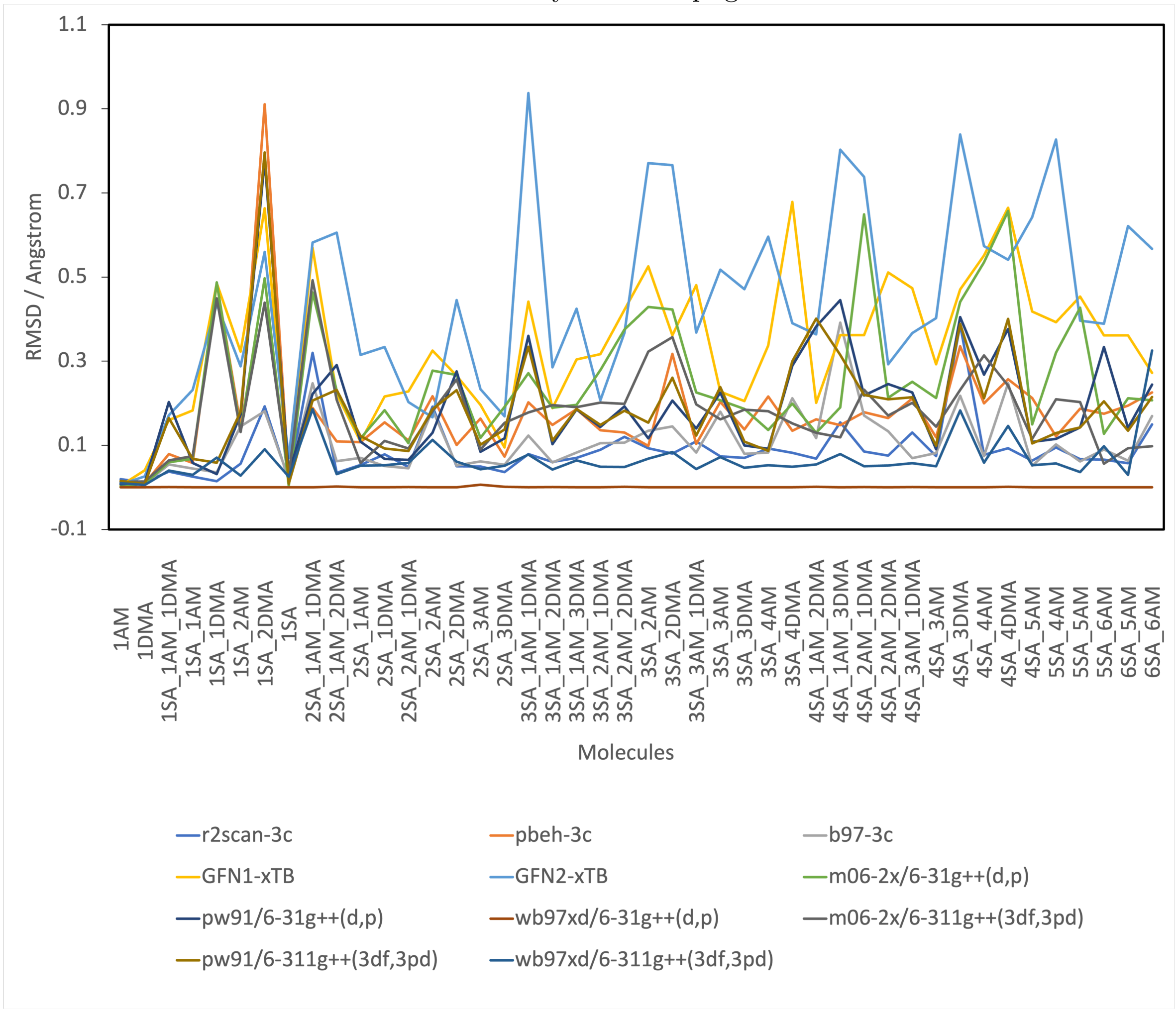

Figure S1: 44 RMSDs of molecules optimized by different methods from the reference geometry.

S2 Parallel ABCluster

In order to test the reliability of ABCluster for obtaining the lowest energy conformers, we ran 100 parallel ABCluster calculations on the $(SA)_{10}(AM)_{10}$ system. These were run using: pop = 1280, gen = 320, scout = 4, and lm = 1280, which gives a CS power of 4.1×10^5. The parallel calculations were optimized using GFN1-xTB and single-point energy corrections were calculated using B97-3c. One of the calculations failed but was not restarted. The resulting distribution of binding free energies can be seen in Figure S2. From Figure S2 (a), it can be seen that the majority of the calculated structures have much higher binding free energy than the lowest free energy structures. The lowest free energy structure is just above −256 kcal/mol, while the distribution peaks around −235 kcal/mol. Figure S2 (b) shows the binding free energy of the lowest obtained energy minimum from each calculation. From this, we can derive the likelihood of obtaining the lowest binding free energy structure if a certain number of runs are performed. The expected error of the B97-3c//GFN1-xTB binding free energy is 3–4 kcal/mol [1], which means that differences smaller than these are not meaningful. Therefore, we define structures within the (−256)–(−252) kcal/mol band to be a 'low-energy' structure. Within this band, we observe 26 structures, and 20 structures if only the (−256)–(−253) kcal/mol band is observed. This means that the probability of obtaining a low-energy structure for a given ABCluster run is between 20–26 %.

The probability of not getting any low-energy structure when only 1 ABCluster run is performed for clusters of this size is therefore 74 %, which is unacceptable. With more parallel runs, this probability is lowered: 2 runs result in 54 %, 5 runs in 22 %, and 10 runs reduce this to 5 %. Therefore, between 5–10 runs is recommended for an acceptable probability of not obtaining the 'low-energy' structure.

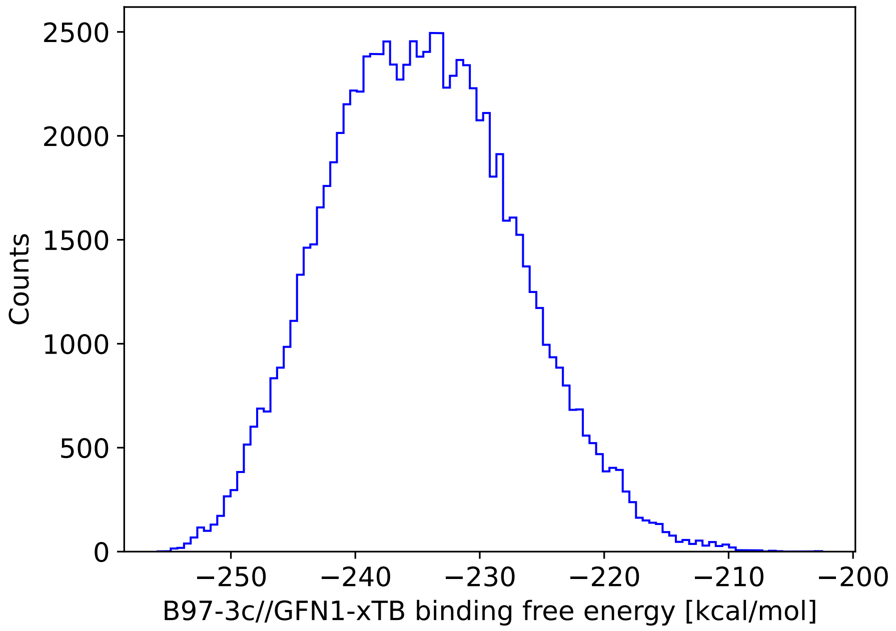

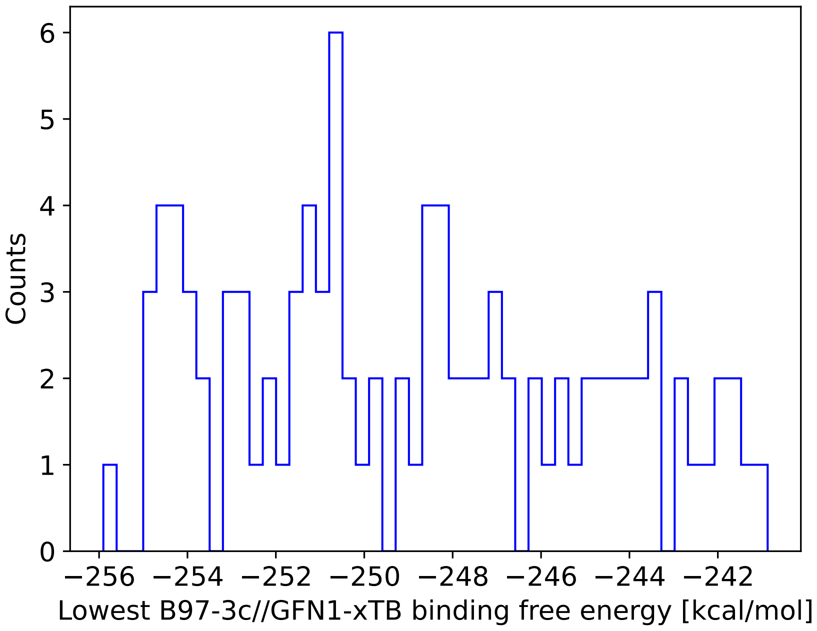

Figure S2: Distributions of structures from 99 ABCluster runs, calculated at the B97-3c//GFN1-xTB level. *(top)* The distribution of all the structures *(bottom)* The distribution of only the lowest energy structure from each parallel run.

S3 Correlation between energy values calculated by GFN1-xTB, r^{2}SCAN-3c and B97-3c

Figure S3 presents the electronic energies of geometries optimized at r^{2}SCAN-3c, B97-3c, and GFN1-xTB and plotted against each other. The most similar results can be found between the r^{2}SCAN-3c and B97-3c methods with a weak correlation ($R = 0.3$).

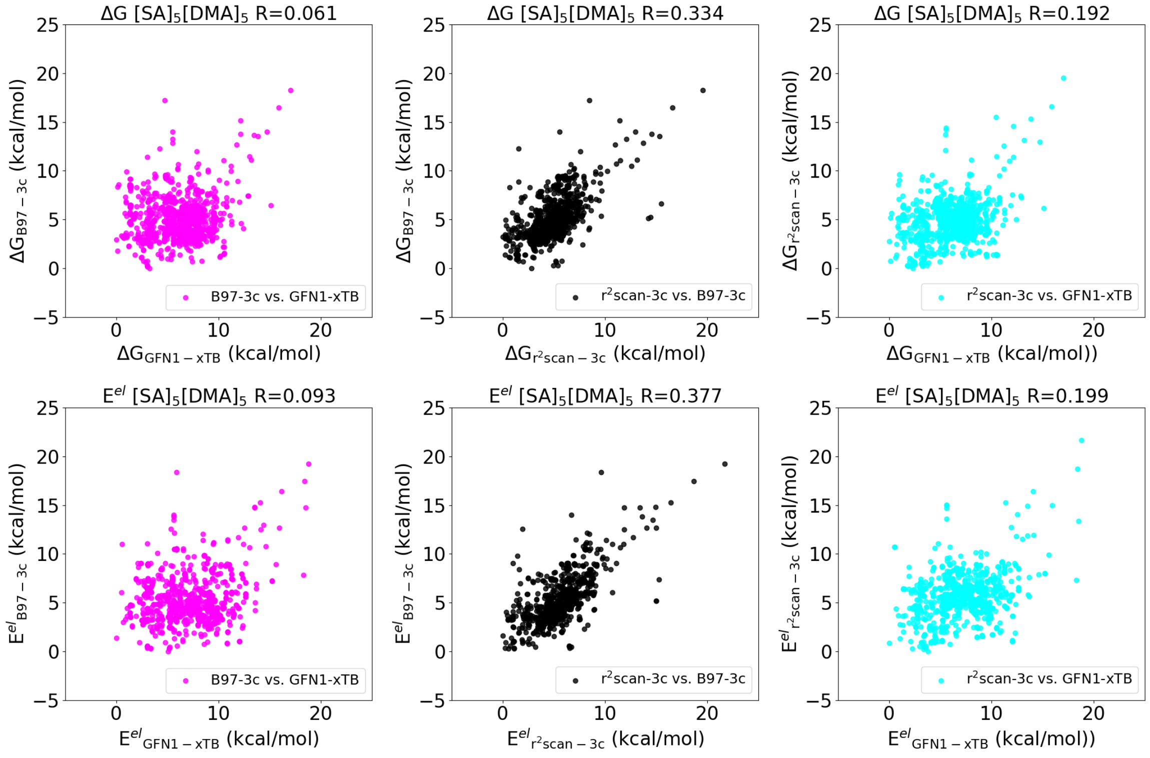

Figure S3: Correlation between the relative electronic/free energies of the $(SA)_5(DMA)_5$ clusters calculated at the GFN1-xTB, r^{2}SCAN-3c, and B97-3c levels of theory.

S4 Geometries from Calculations with and without Pre-optimization

Initiating with the same 30 geometries, calculations were performed with and without a step of pre-optimization at GFN1–xTB level. Afterwards, geometries resulted from the two approaches were compared, and the RMSD distribution is plotted in Figure S4.

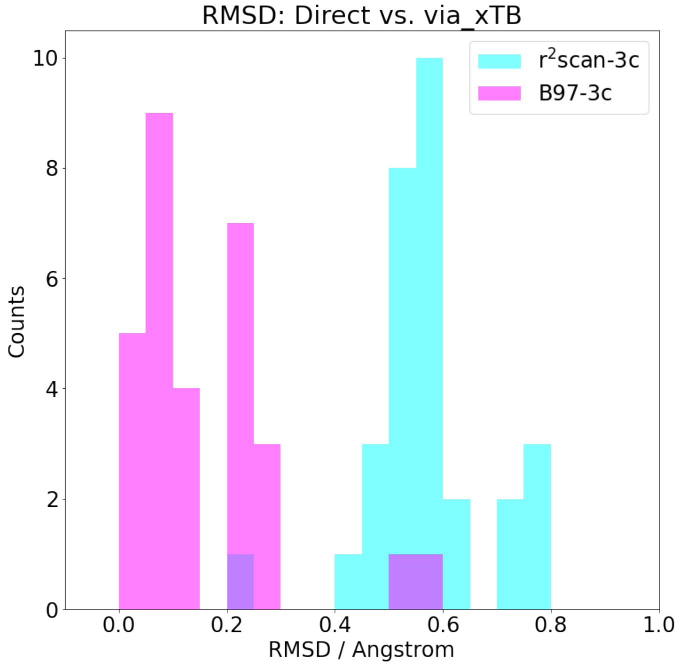

Figure S4: RMSD of $(SA)_{10}(DMA)_{10}$ calculated from the Direct approach vs. via_xTB approach samplings

S5 Possibility of Terminating Geometry Optimization after Certain Iterations

Figure S5 presents 1000 B97-3c optimizations of the $(SA)_5(DMA)_5$ cluster. The 1000 calculations were indexed from 1 to 1000 and sorted by final B97-3c single point energy. After ∼10 iterations, the ordering of the calculations remains constant

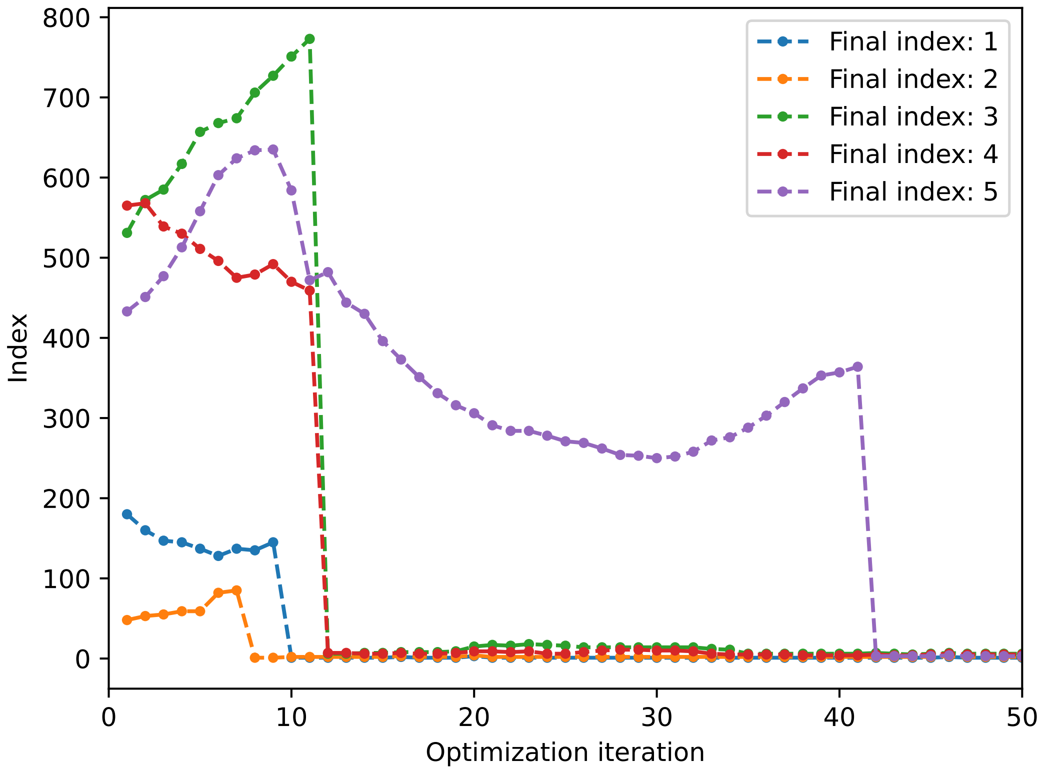

Figure S5: Index of the energy minima as a function of the optimization cycle at B97-3c for the $(SA)_5(DMA)_5$ cluster.

Correlation of the B97-3c single point energies between the B97-3c full geometry optimizations and the partial optimizations (20 iterations).

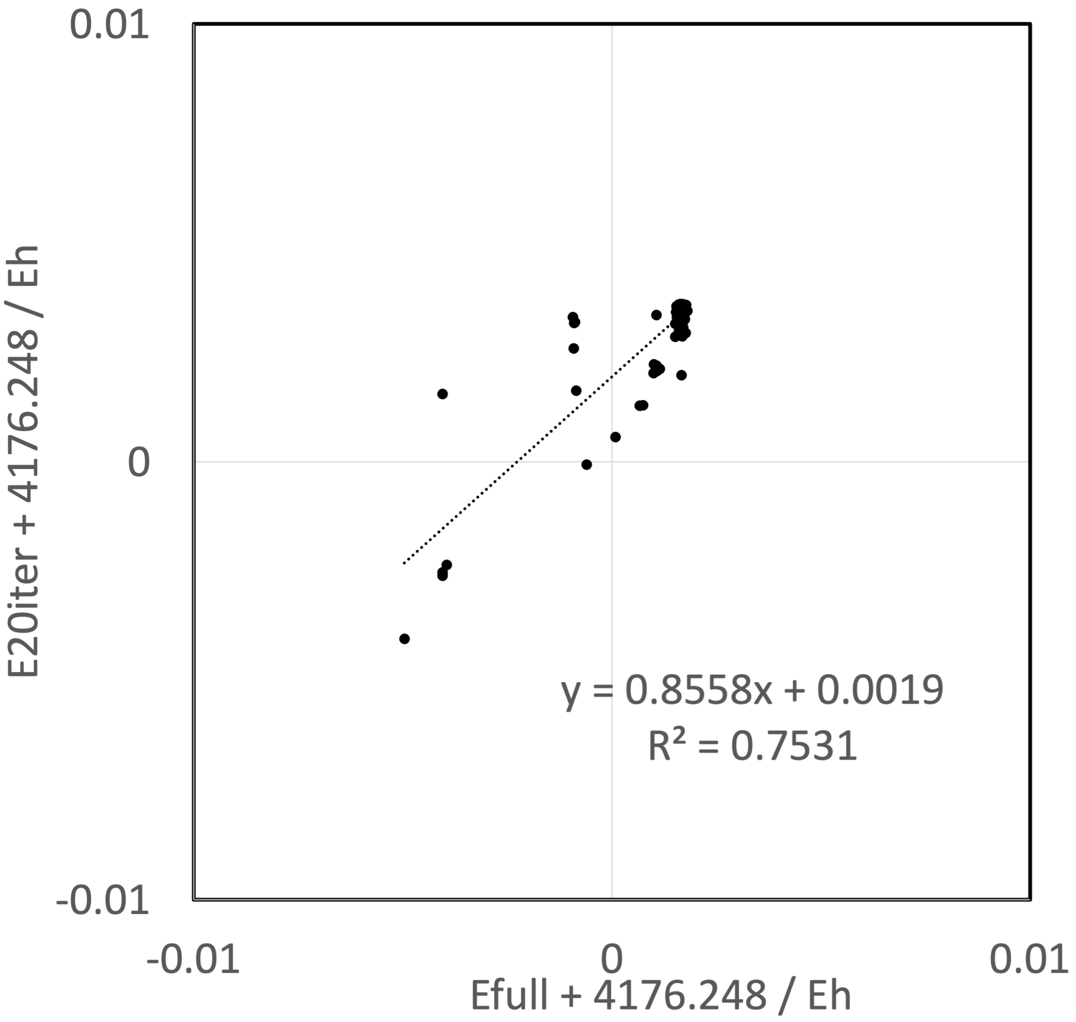

Figure S6: Single-point energies of fully optimized geometries and 20 opt.-iteration geometries of $(SA)_5(DMA)_5$.

S6 Lowest-energy Conformers Found in This Work and Previous Work

n	$\Delta(\Delta G_{\mathrm{SA-AM}})$	$\Delta(\Delta G_{\mathrm{SA-DMA}})$
2	-	-3.8
3	-	-8.1
4	-	-13.9
5	-	-12.7
6	-2.9	-18.8
7	-2.7	-17.8
8	-3.4	-27.2
9	-1.3	-
10	3.6	-
11	-12.9	-
12	-12.2	-
13	-6.5	-
14	-2.9	-
15	-0.6	-

Table S1: Comparison between the previously found global free energy minima by Engsvang et al. and the current study. A negative free energy change shows that the new approach found a better global free energy structure.

References

[1] M. Engsvang and J. Elm, "Modeling the binding free energy of large atmospheric sulfuric acid–ammonia clusters," *ACS Omega*, vol. 7, no. 9, pp. 8077–8083, 2022.
